# Multiple roles for *Plasmodium berghei* phosphoinositide-specific phospholipase C in regulating gametocyte activation and differentiation

**DOI:** 10.1111/j.1462-5822.2011.01591.x

**Published:** 2011-04-26

**Authors:** Andreas C Raabe, Kai Wengelnik, Oliver Billker, Henri J Vial

**Affiliations:** 1 UMR5235, CNRS-Université Montpellier 2, Place Eugène Bataillon34095 Montpellier cedex 5, France; 2 Imperial College London, Division of Cell and Molecular BiologyLondon SW7 2AZ, UK; 3 The Wellcome Trust Sanger InstituteHinxton Cambridge CB10 1SA, UK

## Abstract

Critical events in the life cycle of malaria parasites are controlled by calcium-dependent signalling cascades, yet the molecular mechanisms of calcium release remain poorly understood. The synchronized development of *Plasmodium berghei* gametocytes relies on rapid calcium release from internal stores within 10 s of gametocytes being exposed to mosquito-derived xanthurenic acid (XA). Here we addressed the function of phosphoinositide-specific phospholipase C (PI-PLC) for regulating gametocyte activation. XA triggered the hydrolysis of PIP_2_ and the production of the secondary messenger IP_3_ in gametocytes. Both processes were selectively blocked by a PI-PLC inhibitor, which also reduced the early Ca^2+^ signal. However, microgametocyte differentiation into microgametes was blocked even when the inhibitor was added up to 5 min after activation, suggesting a requirement for PI-PLC beyond the early mobilization of calcium. In contrast, inhibitors of calcium release through ryanodine receptor channels were active only during the first minute of gametocyte activation. Biochemical determination of PI-PLC activity was confirmed using transgenic parasites expressing a fluorescent PIP_2_/IP_3_ probe that translocates from the parasite plasmalemma to the cytosol upon cell activation. Our study revealed a complex interdependency of Ca^2+^ and PI-PLC activity, with PI-PLC being essential throughout gamete formation, possibly explaining the irreversibility of this process.

## Introduction

To be transmitted from the blood stream to a mosquito, malaria parasites rely entirely on highly specialized sexual precursor stages, the gametocytes. While circulating in the blood, mature gametocytes remain in a resting state within erythrocytes, but upon ingestion by a mosquito they rapidly resume development. In response to converging physical and chemical cues from the mosquito midgut environment gametocytes differentiate rapidly into gametes. Activated gametocytes of both sexes emerge from their host erythrocytes and female (macro-) gametocytes are thought to be available for fertilization immediately. Emerged male (micro-) gametocytes, in contrast, require another 10–15 min, during which they enter the cell cycle, complete three cycles of DNA replication and mitosis, assemble axonemes, and then give rise to eight flagellated microgametes in a process termed exflagellation. Gametes fertilize and each zygote then transforms into a motile stage, the ookinete, which from about 20 h post feeding penetrates the mosquito peritrophic matrix and midgut epithelium to establish the infection in the mosquito (Sinden *et al*., 1996; Alano and Billker, 2005). Triggers of gametocyte activation include a drop in temperature, a rise in pH and the small mosquito-derived molecule, xanthurenic acid (Carter and Nijhout, 1977; Nijhout, 1979; Billker *et al*., 1997; 1998; Garcia *et al*., 1997). At a permissive temperature either a rise in pH or xanthurenic acid are sufficient to activate gametocytes (Billker *et al*., 2000). In search of second messengers regulating activation, pharmacological studies identified roles for cyclic guanosine 3′,5′-monophosphate (cGMP) and Ca^2+^ in *P. berghei* and *P. falciparum* (Kawamoto *et al*., 1990). Both pathways were recently confirmed in genetic studies. The only known cGMP effector in *Plasmodium*, protein kinase G (PKG), is essential at an early stage in *P. falciparum* gametocyte activation (McRobert *et al*., 2008).Negative regulation of cGMP in *P. falciparum* gametocytes requires a parasite phosphodiesterase, PDEδ (Taylor *et al*., 2008). In *P. berghei* gametocytes cytosolic Ca^2+^ was measured in a transgenic reporter line expressing a Ca^2+^ sensitive luciferase, which revealed a rapid release of Ca^2+^ from intracellular stores within less than 10 s of exposing gametocytes to xanthurenic acid (Billker *et al*., 2004). In *P. berghei* Ca^2+^ controls all constituent events of gametogenesis, including egress from the host cell, male cell cycle progression and exflagellation. Differentiation of the male gametocyte is regulated through a male-specific Ca^2+^-dependent protein kinase, CDPK4, which is required for the initiation of DNA replication (Billker *et al*., 2004). After replication and mitosis an atypical mitogen-activated kinase-like protein, MAP-2, that serves as substrate for CDPK4 *in vitro*, is then needed at the stage of exflagellation for motile microgametes to emerge (Khan *et al*., 2005; Rangarajan *et al*., 2005; Tewari *et al*., 2005). Both kinases are dispensable for macrogametocyte activation and for gametocyte egress from the host cell in either sex, suggesting other Ca^2+^-dependent events are mediated through different effector pathways.

A parasite receptor for xanthurenic acid has remained elusive and how physical and chemical triggers from the mosquito activate second messenger pathways in gametocytes is largely unknown. In eukaryotes different upstream messengers and channels control Ca^2+^ release from intracellular compartments. One pathway involves ryanodine receptor (RyR) channels on the endoplasmic reticulum (ER), which are bound tightly by the plant alkaloid ryanodine, but which are controlled *in vivo* by the intracellular messenger cyclic ADP ribose (cADPR), the product of a specific cyclase (Galione and Churchill, 2002). *Toxoplasma gondii* can produce cADPR and possesses RyR Ca^2+^ release channels, which regulate intracellular Ca^2+^ in a way that is important for microneme secretion, Ca^2+^-dependent egress and parasite motility (Chini *et al*., 2005; Nagamune *et al*., 2008). Although enzymes and channels involved in cADPR signalling have so far only been identified from animals, at least parts of this pathway seem conserved in Apicomplexa.

Another pathway to Ca^2+^ mobilization relies on phosphoinositide specific phospholipase C (PI-PLC), which hydrolyses the minor membrane lipid phosphatidylinositol-(4,5)-bisphosphate (PIP_2_), producing the secondary messengers inositol-(1,4,5)-trisphosphate (IP_3_) and diacylglycerol (DAG); IP_3_ then triggers Ca^2+^ release into the cytosol by binding to IP_3_-gated Ca^2+^ channels localized predominately in the ER membrane (Berridge *et al*., 2000).

Phosphatidylinositol is the phospholipid that in erythrocytes infected with *P. falciparum* asexual stages experiences the highest relative increase due to biosynthetic activity of the parasite, indicating important biological functions in *Plasmodium* (Vial *et al*., 1990). Parasite-derived PIP_2_ synthesis and Ca^2+^-dependent production of inositol polyphosphates is preponderant in mature asexual blood stage *P. falciparum* parasites (Elabbadi *et al*., 1994). The parasite's PI synthase has been characterized (Elabbadi *et al*., 1994; Wengelnik and Vial, 2007), as has been a phosphatidylinositol 4-phosphate 5-kinase that gives rise to PIP_2_ (Leber *et al*., 2009).

PI-PLC is a strong candidate for regulating cellular Ca^2+^ levels in gametocytes, because IP_3_ and DAG were found previously to increase in response to gametocyte activation in *P. falciparum* (Martin *et al*., 1994). In the current study we examine the role of PIP_2_ hydrolysis during gametogenesis of *P. berghei* in the context of our recent advances in understanding the timing of signalling events in this parasite species. We combine a kinetic analysis with pharmacological experiments to place agonist induced activation of PI-PLC with respect to Ca^2+^ mobilization early in gametocyte activation. We also present evidence for additional roles of IP_3_ production at late stages of gametogenesis.

## Results

### PI-PLC inhibition abolishes gametocyte activation

**Fig. 1:**
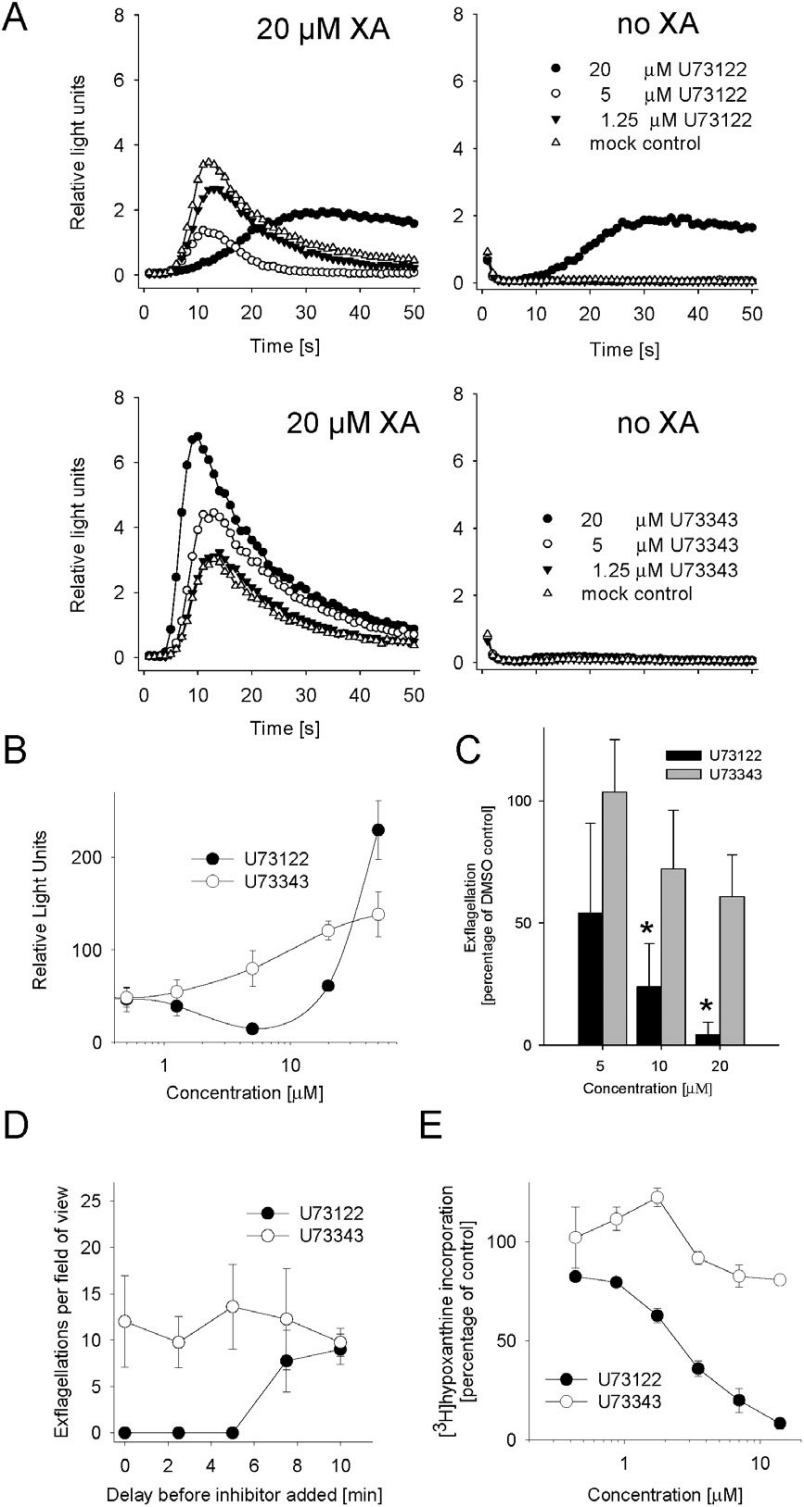
The PI-PLC inhibitor U73122 inhibits Ca^2+^ mobilization, DNA synthesis and exflagellation in *P. berghei* gametocytes. A. Effect of U73122 on light emission over time in gametocytes expressing the Ca^2+^-dependent luciferase GFP–aequorin. Representative time-courses show effects of U73122 (upper panels) and U73433 (lower panels) on XA induced Ca^2+^ mobilization (left) compared with effects of compounds alone (right). XA and compounds were added at time point 0 s. B. Dose-dependent effects of U73122 and U73343 on Ca^2+^-dependent luciferase activity in the presence of 20 µM XA. Relative light units were integrated over the first 50 s after addition of XA + inhibitor. C. Dose-dependent effects on exflagellation of inhibitors added at the moment of microgametocyte activation, expressed as a percentage of a DMSO control. Asterisks indicate significant differences from solvent controls (*P < 0.01, Student's *t*-test). D. Effects on exflagellation of adding 20 µM U73122 or U73343 at different time points after gametocyte activation by XA + pH 8.0. Exflagellation was counted after 12–15 min. Error bars indicate standard deviations among 10 slides from three different experiments. E. Dose-response of U73122 and U73343 for [^3^H]hypoxanthine incorporation as a measure of DNA synthesis during microgametogenesis. Compounds and [^3^H]hypoxanthine were added simultaneously with the activation medium. Error bars in (B), (C) and (E) show standard deviations from 3–4 samples.

In *P. berghei* gametocyte activation requires a rapid increase of cytosolic Ca^2+^ released from intracellular stores, which becomes detectable within 8–10 s of exposing gametocytes to xanthurenic acid at a permissive temperature (Billker *et al*., 2004). Ca^2+^ mobilization in gametocytes can be conveniently measured using a transgenic reporter strain of *P. berghei* that constitutively expresses a Ca^2+^-dependent luciferase, GFP–aequorin. Using this assay we first examined the effect of a widely used inhibitor of PI-PLC dependent signalling, U73122. Between 0.5 and 5 µM U73122 dose-dependently reduced the XA induced Ca^2+^ signal in populations of enriched gametocytes (Fig. 1A), consistent with a role for PI-PLC upstream of Ca^2+^ mobilization. However, at 20 µM U73122 we unexpectedly observed an increase in cytosolic Ca^2+^, albeit with a time-course atypical of an XA-induced response (Fig. 1A, left). In fact, at this concentration, U73122 mobilized intracellular Ca^2+^ independently of XA (Fig. 1A, right). We next compared U73122 with its inactive structural analogue, U73343. In Fig. 1B the total luciferase activity during the first 50 s after XA activation is plotted against compound concentration, showing that inhibition of the XA-induced Ca^2+^ response was specific to U73122 and maximal at around 5 µM. The ‘inactive’ analogue did not reduce the Ca^2+^ signal but instead enhanced the XA-induced Ca^2+^ response (Fig. 1A lower panels and Fig. 1B). The selective inhibitory effect of U73122 over its structural analogue would be consistent with an early role for PI-PLC during the first few seconds of gametocyte activation, and upstream of Ca^2+^ release. Consistent with this hypothesis, 20 µM U73122 inhibited exflagellation completely and selectively over U73343 (Fig. 1C). We next asked whether the addition of inhibitor at different time points after the initial Ca^2+^ burst would still block exflagellation. Exflagellation remained sensitive to U73122 when the inhibitor was added to the gametocyte culture at any time during at least the first 5 min after activation, but thereafter became resistant (Fig. 1D). This indicates that PI-PLC activation is required beyond the first few seconds of gametocyte activation, during which intracellular Ca^2+^ is mobilized. The resistance of activated gametocytes after 5 min furthermore shows that neither U73122 nor U73343 exhibited non-specific toxicity towards gametocytes. Exflagellation is a highly dynamic process and inherently difficult to quantify. A more robust measure of male gametocyte activation can be obtained from a [^3^H]hypoxanthine incorporation assay, which determines DNA synthesis during the rapid threefold genome replication that precedes microgamete release (Raabe *et al*., 2009). We used this assay to determine the IC_50_ of U73122 as being just below 3 µM (Fig. 1E). This inhibitor thus blocks gametocyte activation selectively over U73343 at the same concentration, at which rapid Ca^2+^ mobilization within the first 10 s is also inhibited (Fig. 1A).

### Changes of PI-PLC substrate levels upon gametocyte activation

**Fig. 2:**
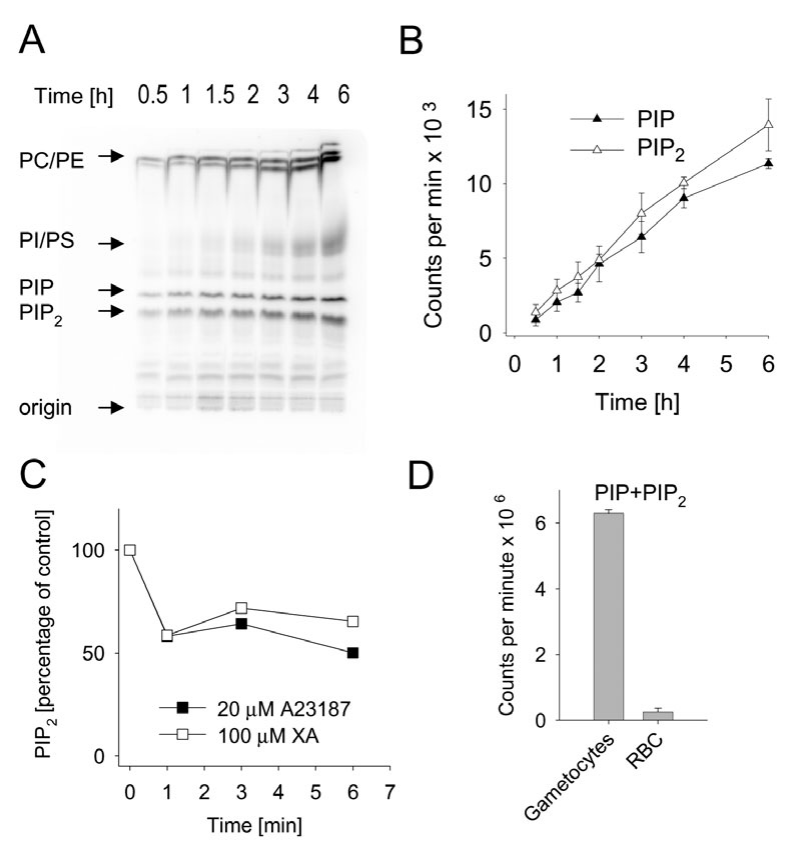
PIP_2_ hydrolysis during gametogenesis. A. Phosphoimager scan of a TLC plate showing separation of phospholipids from 9.2 × 10^7^ purified gametocyte-infected erythrocytes labelled with [^32^P] orthophosphate for 0.5–6 h. The position of lipid standards is indicated. PI, phosphatidylinositol; PS, phosphatidylserine; PC, phosphatidylcholine; PE, phosphatidylethanolamine. B. PIP and PIP_2_ bands were quantified by liquid scintillation counting and plotted against labelling period. Error bars show SE of two experiments. C. PIP_2_ levels in gametocytes treated with XA or A23187 after previous labelling with ^32^P for 90 min, expressed as a percentage of time-matched mock treated controls. Shown is one representative experiment of three. D. ^32^P incorporation into PIP + PIP_2_ by gametocyte-infected and uninfected erythrocytes labelled for 6 h. Error bars show standard deviations in duplicate measurements from two independent experiments.

We next sought to measure the cellular PI-PLC activity in intact cells directly by monitoring the level of radiolabelled cellular PIP_2_, the substrate of PI-PLC. Incubating preparations of highly enriched gametocytes with [^32^P]orthophosphate resulted in efficient incorporation of radiolabel into PIP, PIP_2_ and other phospholipids, as revealed by thin layer chromatography (TLC) of extracted cellular lipids in parallel with lipid standards (Fig. 2A). Label incorporation into phosphoinositides was linear over a 6 h incubation period (Fig. 2B), and male gametocytes retained their ability to differentiate into gametes for up to 3 h of culture *in vitro* (data not shown). We therefore routinely assayed PI-PLC activity after 2 h of labelling, when gametocytes were still unaffected in their ability to differentiate. When gametocytes were activated by XA, PIP_2_ levels decreased within the first minute (Fig. 2C) and then remained depressed if compared with time-matched, mock treated control cells. The Ca^2+^ ionophore A23187 produced a similar drop in cellular PIP_2_ levels, consistent with the ability of Ca^2+^ to activate PI-PLC in *P. falciparum* infected erythrocytes (Elabbadi *et al*., 1994). We wondered if the PIP_2_ hydrolysis we observed could be attributed entirely to the parasite, or if some occurred in the host cell compartment. However, at room temperature [^32^P]orthophosphate incorporation into uninfected erythrocytes was only 4% of gametocyte infected cells (Fig. 2D). Host cell phosphoinositides are thus unlikely the make a significant contribution to the PIP_2_ hydrolysis shown in Fig. 2C.

### Analysis of PI-PLC product levels upon gametocyte activation

**Fig. 3:**
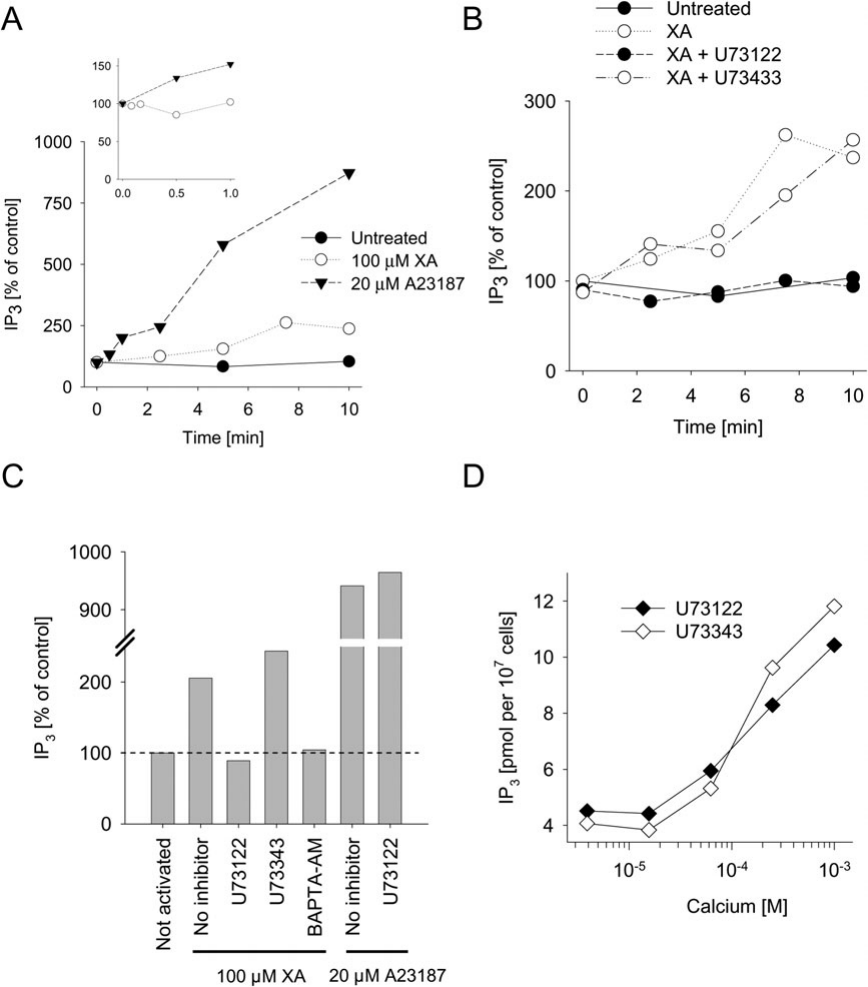
IP_3_ production during gametogenesis. A. IP_3_ content of purified gametocyte-infected erythrocytes at different times after treatment with XA, A23187 or solvent control, expressed as a percentage of the resting level (around 2 pmol per 10^7^ gametocytes). Inset shows immediate onset of IP_3_ production only in A23187 treated cells. Shown is a representative result from two experiments. B. Effect of U73122 and U73343 (both 10 µM) on IP_3_ following activation by 100 µM XA. C. Effect of inhibitors on cellular IP_3_ content 10 min after treatment with either XA or A23187. D. Effect of various Ca^2+^ concentrations in the culture medium on IP_3_ content 10 min after ionophore activation (20 µM A23187) in the presence of either 10 µM U73122 or 10 µM U73343.

In complementary experiments we also determined the level of IP_3_, the product of PIP_2_ hydrolysis, using a Biotrak assay system. XA-independent PI-PLC activation by Ca^2+^ ionophore A23187 resulted in a rapid and sustained increase of total IP_3_ levels in gametocyte cultures (Fig. 3A). In contrast, gametocyte activation by XA produced a marked but weaker and more delayed response, in which a rise in IP_3_ did not become apparent until later than one minute of activation (Fig. 3A, inset). Importantly, XA-induced IP_3_ production continued throughout gametocyte differentiation (Fig. 3B). The XA-induced rise in cellular IP_3_ was totally abolished by U73122, but not U73343 (Fig. 3B and C), consistent with PI-PLC being involved. XA-induced IP_3_ production was completely inhibited by the membrane permeable Ca^2+^ chelator, BAPTA-AM (Fig. 3C). PI-PLC thus appears to require cellular Ca^2+^. Surprisingly, however, PI-PLC activity was not sensitive to U73122 when activated by the Ca^2+^ ionophore A23187 (Fig. 3C). We hypothesized that unphysiologically high Ca^2+^ levels could overcome PI-PLC inhibition by U73122. However, when we varied extracellular Ca^2+^ over a wide range of concentrations before adding the ionophore, we failed to find a condition at which Ca^2+^-induced IP_3_ production was selectively inhibited by U73122 over U73343 (Fig. 3D). We conclude that direct activation of PI-PLC though Ca^2+^ may bypass inhibition by U73122, which has an unknown mechanism of action.

### Single cell imaging using a PIP_2_/IP_3_ binding fluorescent reporter protein

**Fig. 4:**
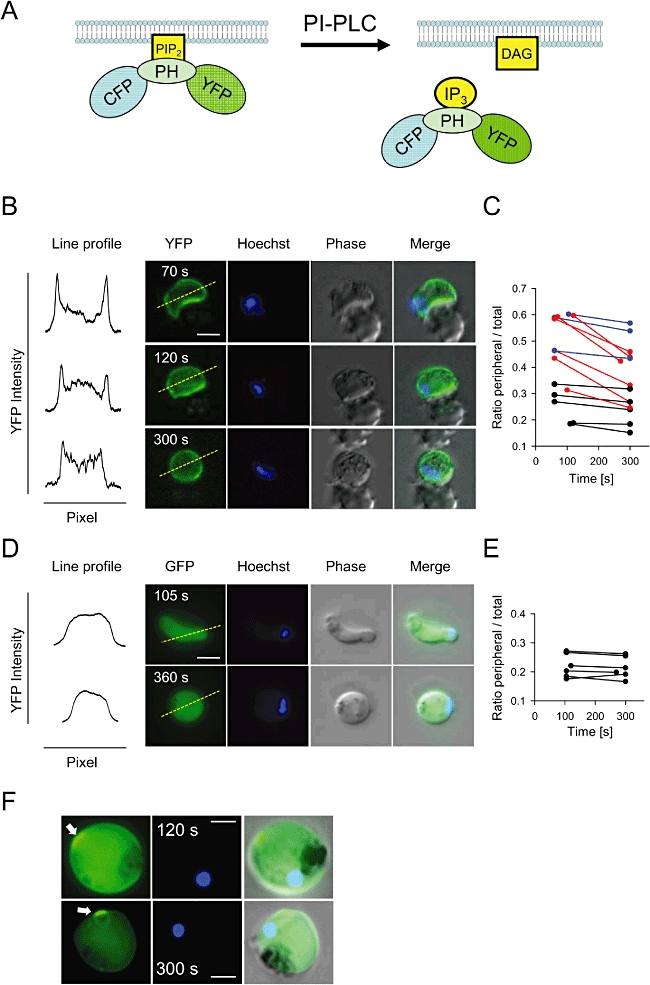
A fluorescent reporter protein to monitor PI-PLC activation in single live *P. berghei* gametocytes. A. Scheme illustrating translocation from the plasma membrane to the cytosol of the CFP–PH–YFP protein upon PI-PLC activation. B. Light microscopic images of a CFP–PH–YFP expressing macrogametocyte recorded at different time points after activation with XA. The first column shows the line profiles of the intensity of YFP fluorescence along the dashed lines in the corresponding YFP images. C. CFP–PH–YFP redistribution in 14 randomly selected macrogametocytes. See text for an explanation of line colours. D. Time lapse microscopy and intensity profiles after activation of macrogametocytes expressing GFP without a PH domain as a control probe. E. Absence of peripheral localization and redistribution of GFP lacking a PH domain. F. Fluorescence images of typical CFP–PH–YFP expressing macrogametocytes showing a disc-like structure (arrows) seen in the majority of gametocytes after rounding up. All indicated times are seconds post activation. The scale bar is 2 µm in all panels.

We next sought to observe PI-PLC activation at the level of the individual gametocyte. Dynamic changes in cellular PIP_2_ have been monitored successfully in cultured mammalian cells by single cell imaging of a fluorescent reporter protein fused to the PH domain of human phospholipase Cδ1 (hPLCδ1) (Violin *et al*., 2003). PH domains can bind both, PIP_2_ and IP_3_. Resting cells contain low IP_3_ levels and a PH domain-containing reporter protein is targeted mostly to the plasma membrane where PIP_2_ resides. PI-PLC activation and IP_3_ production then leads to translocation of the probe to the cytoplasm (Fig. 4A). We generated a *P. berghei* expression cassette, in which the strong constitutive *ef1α* promoter controls expression of a fusion protein consisting of the PH domain of hPLCδ1 fused to yellow fluorescent protein (YFP) and cyan fluorescent protein (CFP). A vector containing this reporter cassette, together with a *Tgdhfr/ts* selection marker for antimalarial drug resistance, was then introduced into *P. berghei* schizonts by electroporation, and maintained as episome by selecting for the resistance marker. In most resting gametocytes of either sex the CFP–PH–YFP protein was clearly detectable in the periphery of the cells, consistent with a localization at the plasma membrane (Fig. 4B). By time lapse microscopy we observed that within two minutes of activation by XA, the CFP–PH–YFP protein began to redistribute to the cytosol, a process that was typically complete 5 min after gametocyte activation (Fig. 4B). A quantitative analysis in randomly selected macrogametocytes found that CFP–PH–YFP redistributed to the cytosol in about half of the cells (red lines in Fig. 4C). A few cells showed a high proportion of peripherally located CFP–PH–YFP that did not change upon addition of XA (blue lines in Fig. 4C); these cells may have been immature gametocytes still unable to respond to XA. Other gametocytes had a relatively high level of cytosolic fluorescence that remained unchanged (black lines in Fig. 4C). The latter response was typical of the cytosolic localization in the control cell line expressing GFP without a PH domain (Fig. 4D and E). CFP–PH–YFP expressing cells with cytosolic localization of the marker may have responded already during the minute that typically elapsed between gametocyte activation and recording of the first image. Male and female gametocytes both showed redistribution of the CFP–PH–YFP reporter constructs, but due to the choice of promoter the reporter protein was more strongly expressed and easier to detect in macrogametocytes (not shown). In the vast majority of gametocytes CFP–PH–YFP accumulated transiently in a disc-like structure in the cell periphery (Fig. 4F). We have no explanation for this structure, but believe it may indicate a transient heterogeneity in membrane lipid composition of differentiating gametocytes that could be linked to the marked changes in cell shape and volume during gametogenesis (Sinden and Croll, 1975).

### Analysis of RyR channels in Ca^2+^ release

**Fig. 5:**
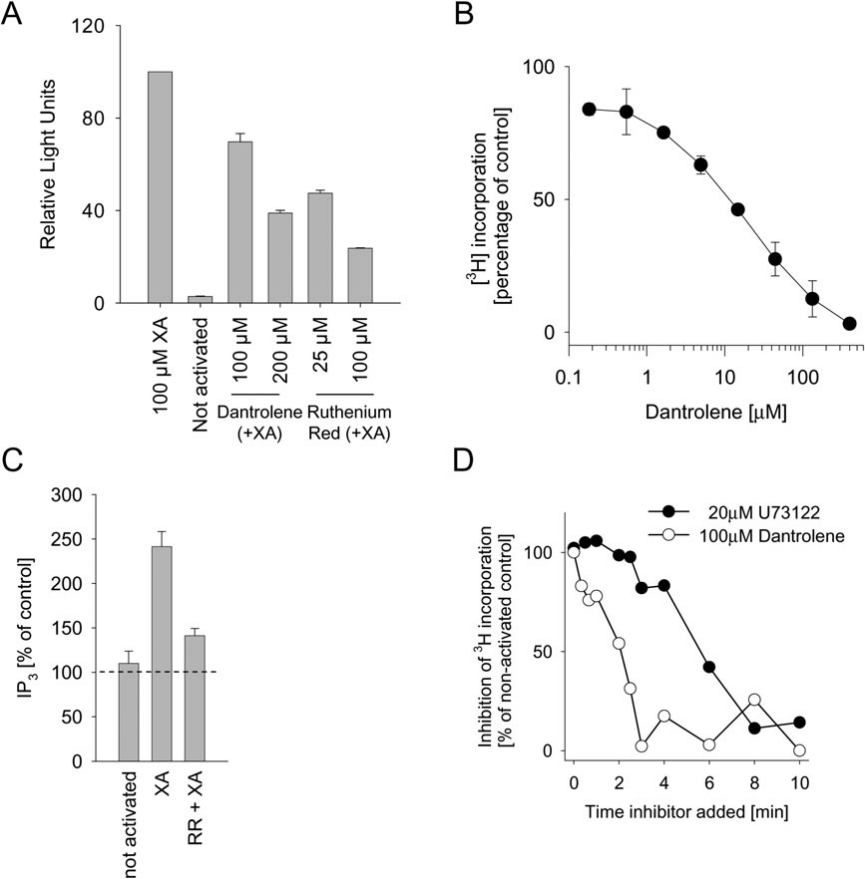
RyR receptor channel antagonists inhibit Ca^2+^ mobilization and IP_3_ production upon gametocyte activation. A. Effect of dantrolene and ruthenium red on induction of Ca^2+^ mobilization by 100 µM XA, measured as light emitted over 50 s in GFP–aequorin expressing gametocytes. Error bars show standard deviations (*n* = 4). B. Effect of dantrolene on microgametogenesis, measured as [^3^H]hypoxanthine incorporation into DNA. Error bars show standard deviations (*n* = 3). C. Effect of 100 µM ruthenium red on IP_3_ produced by gametocyte-infected erythrocytes activated by 100 µM XA or mock treatment (pipetting). IP_3_ is given as a percentage of untreated control cells. Error bars show standard deviations (*n* = 4). Inhibitors in panels A-C were added at the time of gametocyte activation by XA. D. Effect of delayed addition of agents on inhibition of microgametogenesis, as measured by DNA synthesis ([^3^H]hypoxanthine incorporation) during the first 20 min of activation. Dantrolene was used at 100 µM, U73122 at 20 µM. Inhibitors were added after 100 µM XA-induced onset of gametogenesis at indicated time points. The inhibition of label incorporation is expressed relative to non-activated control cells. Shown is one representative experiment of three.

In many mammalian tissues IP_3_ receptor channels coexist and interact with Ca^2+^ release through RyR channels, for instance during Ca^2+^-induced Ca^2+^ release (Berridge *et al*., 2000; 2003;). In view of the importance of RyR in agonist-induced signalling in the closely related parasite *Toxoplasma gondii*, we examined two inhibitors that have been validated for blocking RyR in this species, dantrolene and ruthenium red (Chini *et al*., 2005; Nagamune *et al*., 2008), in *P. berghei* gametocytes. Both compounds significantly inhibited the rapid Ca^2+^ response to XA (Fig. 5A) and also inhibited exflagellation (data not shown), an effect we quantified in the [^3^H]hypoxanthine incorporation assay for dantrolene (Fig. 5B). Reduced IP_3_ production in gametocytes treated with ruthenium red (Fig. 5C) indicated that RyR mediated Ca^2+^ mobilization may be required for sustained PI-PLC activity during gametogenesis, not only for the early agonist mediated Ca^2+^ burst. Having shown that exflagellation remained sensitive to PI-PLC inhibition by U73122 even beyond the initial XA-induced Ca^2+^ burst (Fig. 1D), we wondered whether a RyR channel antagonist would be more selective for early events leading to rapid Ca^2+^ mobilization. To examine this possibility we added either dantrolene or U73122 at different time points after activation, and asked when [^3^H]hypoxanthine incorporation became insensitive to the inhibitors (Fig. 5D). The window of sensitivity differed markedly between both compounds. Dantrolene only exerted its full inhibitory effect when added simultaneously with XA. In contrast, gametocyte differentiation remained sensitive to addition of U73122 for an extended period of 4–6 min after activation. Taken together, these data suggest that ryanodine receptors and IP_3_-mediated mechanisms are likely to be responsible for the rapid early Ca^2+^ mobilization in activated gametocytes. In contrast, in order for gametogenesis to be completed sustained activation of PI-PLC for a more extended period, resulting in increasing IP_3_ levels, is required.

## Discussion

Ca^2+^ is an important second messenger regulating key events throughout the life cycle of apicomplexan parasites (Billker *et al*., 2009). In *Plasmodium* the activation of gametocytes currently provides the best documented example of a signal transduction pathway leading from extracellular signals, via the rapid release of Ca^2+^ from intracellular stores, to a stage-specific Ca^2+^ effector pathway for cellular differentiation (Billker *et al*., 2004). How extracellular signals are linked to Ca^2+^ release in malaria parasites is an important question that is difficult to address experimentally, since candidate genes for signalling receptors have not been identified in *Plasmodium*. To help close this gap we have here investigated the role for PI-PLC in XA induced activation of gametocytes in *P. berghei*.

All malaria species encode in their genome a single candidate gene for PI-PLC (e.g. *P. falciparum* PF10_0132 and, *P. berghei* PBANKA_121190), which is characterized by a predicted domain organization largely conserved from human PLCδ isoforms to yeast PI-PLC (Williams and Katan, 1996). Like its *T. gondii* orthologue (Fang *et al*., 2006), *Plasmodium* PI-PLC has a predicted N-terminal Pleckstrin homology (PH) domain presumably required for targeting PI-PLC to the plasma membrane, and a bipartite catalytic domain flanked by Ca^2+^-binding EF hands and a C2 domain that could be involved in binding to membrane phospholipids in a Ca^2+^-dependent or independent manner. Consistent with this conserved domain organization, recombinant *Tg*PI-PLC, like PI-PLC isozymes from other organisms, is strictly Ca^2+^-dependent *in vitro* (Fang *et al*., 2006).

Measuring different inositol phosphates after metabolic labelling with [^3^H]*myo-*inositol, Martin *et al*. (1994) demonstrated that IP_3_ is produced in activated *P. falciparum* gametocytes and showed that it can be degraded via two routes: dephosphorylation to yield Ins(1,4)P_2_ or phosphorylation to yield Ins(1,3,4,5)P_4_. All our biochemical and functional assays clearly establish that PI-PLC activity is stimulated upon gametocyte activation by XA in *P. berghei*. We find that levels of PIP_2_ drop within a minute of activation and thereafter remain below resting level (Fig. 2C). An increase in IP_3_ becomes measurable with some delay, but then high levels of IP_3_ persist for up to 20 min (Fig. 3B and data not shown). A continuous rise in IP_3_ levels reflects well the persistent reduction of PIP_2_ levels below baseline, suggesting continuous activity of PI-PLC, as in *P. falciparum*. The kind of CFP–PH–YFP probe that we used here binds both PIP_2_ and IP_3_ and has been described to translocate to the cytoplasm more as a consequence of rising IP_3_ levels than of decreasing PIP_2_ levels at the plasma membrane (Hirose *et al*., 1999). The time-course we report here for the cytosolic translocation of the PH-YFP probe 3–5 min after activation (Fig. 4B) also seems to mirror the increase in IP_3_ (Fig. 3A) better than the decrease in PIP_2_ levels (Fig. 2C).

Gametocyte PIP_2_ is clearly not depleted entirely upon activation (Fig. 2C), as it would be in some mammalian model systems (Suh and Hille, 2007). Why is a large proportion of PIP_2_ not hydrolysed? We show that erythrocytes hardly incorporate [^32^P]orthophosphate into phosphoinositides at room temperature, and thus a major contribution of host lipids to our PIP_2_ measurements is unlikely. We have used a fluorescent probe that reports specifically on PI-PLC activity in the parasite cytosol and the data are consistent with overall changes in lipid composition upon gametocyte activation being primarily due to changes in the parasite. It remains a theoretical possibility, however, that a *Plasmodium* infection activates host cell PI-kinases in the red blood cell cytoplasm resulting in elevated PIP_2_ levels that would be inaccessible to the parasite's PI-PLC enzyme. It has been published that in mature human erythrocytes a calcium-dependent ‘phosphoinositidase C’ activity can be induced upon treatment with an ionophore (Gascard *et al*., 1989). A physiological trigger, however, and the function of the reaction products DAG and IP_3_ have, to our knowledge, not been identified, and common downstream effectors like the IP_3_-receptor and protein kinase C are thought to be absent from erythrocytes. Alternatively, if a large proportion of gametocytes were non-responsive to XA, this could explain why more than half of the labelled PIP_2_ appears unhydrolysed upon gametocyte activation. However, this is also unlikely since PI-PLC in such non-responsive gametocytes would presumably still be activated by the Ca^2+^ ionophore. We find it most likely that PIP_2_ is re-synthesized at a rate similar to its hydrolysis. Consistent with this hypothesis, we find the IP_3_ levels keep rising throughout gametogenesis, which requires PIP_2_ to be replenished. The last step in PIP_2_ biosynthesis is catalysed by a phosphatidylinositol 4-phosphate 5-kinase (PIP5K). This enzyme has been characterized in *P. falciparum* (Leber *et al*., 2009) as part of a putatively bifunctional protein contain N-terminally EF-hand-like motifs found in a family of neuronal Ca^2+^ sensor (NCS) proteins. It has been suggested that Ca^2+^ sensor domains could regulate PfPIP5K activity, linking directly cytosolic Ca^2+^ to PIP_2_ synthesis (Leber *et al*., 2009). This might lead to enhanced PIP_2_ synthesis and could be crucial for the sustained IP_3_ production we observe following the initial Ca^2+^ release after gametocyte activation. Alternatively, incomplete PIP_2_ hydrolysis could result from a partly inaccessible PIP_2_ pool. It is intriguing to speculate that the disk-like peripheral structure, in which the PIP_2_ binding PH-YFP reporter protein accumulates transiently after gametocyte activation (Fig. 4B and F), could be a specialized membrane domain or compartment, in which PIP_2_ is protected from hydrolysis.

The pharmacology of PI-PLC is still poorly understood. Our attempts to produce recombinant *Plasmodium* PI-PLC protein have been unsuccessful, preventing biochemical characterization of purified enzyme. We have indications that deletion or overexpression of the PI-PLC gene is deleterious for *P. berghei* blood stage development (A.C. Raabe, O. Billker, K. Wengelnik, unpublished) excluding genetic approaches. Thus, we rely on pharmacology to place PI-PLC activity with respect to agonist-induced Ca^2+^ mobilization during gametocyte activation. We find that Ca^2+^ release in gametocytes is selectively inhibited by U73122 over its structural analogue, U73343, placing PI-PLC upstream of the rapid calcium release. The aminosteroid U73122 and its control compound are used widely to infer PI-PLC in signalling processes, but some studies have also reported significant off-target effects (Horowitz *et al*., 2005 and references therein). Consistent with this we find that U73122 and its ‘inactive’ analogue, U73343, can both non-selectively facilitate the mobilization of Ca^2+^ release in gametocytes at concentrations only just above those at which U73122 selectively inhibits XA-induced calcium release (Fig. 1B). The molecular mechanism of inhibition of PI-PLC by U73122 remains controversial. Some studies report direct inhibition of catalytic activity by U73122 of recombinantly expressed PLC isozymes (Staxen *et al*., 1999). However, other PI-PLC enzymes, including that from *T. gondii*, are not inhibited *in vitro* (Fang *et al*., 2006). We find that Ca^2+^/ionophore-activated PI-PLC is resistant to U73122, suggesting the compound does not target the catalytic site but interferes in some other way with enzyme activation in intact cells. Nevertheless, U73122 in *Plasmodium* is clearly able to uncouple PI-PLC from its natural upstream activators.

The additional ability of U73122 to mobilize gametocyte Ca^2+^ (Fig. 1A) at 20 µM probably relies on a different mechanism that is independent of PI-PLC, since at the same high concentration IP_3_ production is effectively inhibited (Fig. 3B). The highly lipid-soluble and chemically reactive U73122 cation may exert its non-specific effects by sequestering membrane lipids, or by covalently modifying membrane proteins (Horowitz *et al*., 2005), which could explain the complex results some investigators have obtained with this compounds (Mogami *et al*., 1997). We have therefore used one functional and two biochemical assays to demonstrate that U73122 inhibits IP_3_ production selectively over its control compound, U73343, and that at the appropriate concentration this inhibition is strictly correlated with a block in Ca^2+^ release, and gametocyte differentiation.

That PI-PLC plays a key role early in gametocyte activation, at the time of rapid Ca^2+^ release, is supported by the selective inhibitory effect of U73122 (Fig. 3B and C), by our observation that PIP_2_ hydrolysis is initiated during the first minute of activation (Fig. 2C) and by previous evidence that in *P. falciparum* gametocytes IP_3_ levels shoot up within 30 s of activation (Martin *et al*., 1994). In *P. berghei* accumulation of IP_3_ appeared to trail PIP_2_ hydrolysis, becoming measurable only from 2 min after activation (Fig. 3A and B), although both might be expected to reflect PI-PLC activity. We can only speculate that an initial rapid increase in IP_3_ may be too small to become detectable by the assays we used, or that the first burst of IP_3_ may be rapidly degraded or metabolized to more highly phosphorylated inositol phosphates.

Which function PI-PLC might have during late stages of gametogenesis is unclear. Is it required to keep Ca^2+^ levels elevated? Our GFP–aequorin reporter assay is optimized to detect the initial Ca^2+^ release with exquisite sensitivity (Billker *et al*., 2004). However, it is unable to measure absolute Ca^2+^ levels reliably, due to the rapid depletion of the luciferase-substrate complex. The use of membrane permeable fluorescent Ca^2+^ sensor dyes (Garcia *et al*., 1996) could overcome this limitation. However, in our hands these dyes proved impractical in gametocytes since they were either hardly incorporated, showed high bleaching rates, or lead to exflagellation without XA stimulation (A.C. Raabe, K. Wengelnik, unpublished). We therefore do not know whether sustained IP_3_ production results in constantly elevated levels of cellular free Ca^2+^. It is also unknown whether diacylglycerol, the other product of PIP_2_ hydrolysis, has a signalling role in *Plasmodium*. A major target for DAG in other eukaryotes is protein kinase C, which has no obvious orthologue in Apicomplexa.

While on the one hand IP_3_ thus appears to be required for Ca^2+^ release, Ca^2+^ may in turn enhance PI-PLC activity. In support of this we found that a Ca^2+^ ionophore is sufficient to trigger activation of PI-PLC in resting *P. berghei* gametocytes in an XA-independent manner (Figs 2C and 3A), and that XA-mediated activation of PI-PLC is prevented when cytosolic Ca^2+^ is chelated by BAPTA-AM (Fig. 3C). As is typical of PLCδ isoforms, recombinant *T. gondii* PI-PLC was shown to be Ca^2+^-dependent (Fang *et al*., 2006). We were unable to express recombinant *Plasmodium* PI-PLC to confirm this, but Ca^2+^ binding C2 and EF hand domains appear to be intact in its conserved sequence.

We were intrigued to find that the RyR antagonists dantrolene and ruthenium red also inhibit early Ca^2+^ release and PI-PLC activation. The natural RyR agonist cADPR may thus be involved in the initial mobilizationof Ca^2+^, which could then trigger or support the more sustained activation of PI-PLC, resulting in Ca^2+^ release through IP_3_ receptor channels and irreversible activation of the gametocyte. Consistent with this model we find that male gametogenesis started to become insensitive to dantrolene within seconds of activation, but remained sensitive to U73122 for a much longer period (Fig. 5D). It is tempting to speculate that, following RyR activation during the initial rise of Ca^2+^ levels, positive feedback regulation of PI-PLC and Ca^2+^ could become important for gametocyte activation and for the continuous production of IP_3_ throughout gametogenesis. The likely irreversible nature of this process may be one reason why the initiation of gametogenesis needs to be so well controlled by multiple converging environmental factors.

Work in *T. gondii* has demonstrated that both IP_3_ and cADPR can trigger the release of Ca^2+^ from ER-derived membrane microsomes *in vitro*, and has validated in an apicomplexan parasite the use of ruthenium red and dantrolene as compounds that selectively block RyR (Chini *et al*., 2005). Both pathways may be involved in regulating gliding motility (Chini *et al*., 2005). More recently abscisic acid, previously known only as a plant hormone, was discovered to be an endogenously produced inducer of cADPR production, leading to tachyzoite egress (Nagamune *et al*., 2008). It will be interesting to investigate whether *Plasmodium* gametocytes posses an ADP-ribosyl cyclase activity that is stimulated by XA. Genes encoding IP_3_ receptors, RyR receptors or a ADP-ribosyl cyclase to produce cADPR have so far only been identified in animals and no obvious homologues are present in apicomplexan genomes (Billker *et al*., 2009). In mammalian cells activation of PI-PLC relies on heterotrimeric G-proteins or phosphorylating receptors (Rebecchi and Pentyala, 2000), which also appear to be absent from Apicomplexa. The identification of novel receptor mechanisms that link extracellular signals to Ca^2+^ release in *Plasmodium* will therefore be a major challenge for future research.

## Experimental procedures

### Solutions and chemicals

All chemicals were purchased from Sigma (France) unless otherwise stated. Stock solutions for dantrolene (10 mM), U73122 (2 mM), U73343 (2 mM), A23187 (4 mM), 8-Br-A23187 (4 mM) were made up in DMSO. As dantrolene is reportedly light sensitive, it was prepared fresh for each experiment. The final concentration of DMSO in all assays did not exceed 1%, a concentration that does not inhibit exflagellation or Ca^2+^ mobilization in gametocytes. Stock solutions for ruthenium red (10 mM) and xanthurenic acid (10 mM) were made up in water. Radioactive hypoxanthine was purchased from GE Healthcare, France ([^3^H]hypoxanthine stock solution: 52 µM hypoxanthine in water/ethanol 1:1; specific activity 1 mCi ml^−1^). Radioactive phosphate was purchased from Perkin Elmer ([^32^P] phosphorus as H_3_[^32^P]O_4_, 5 mCi ml^−1^ in water with specific activity of 285.6 Ci mg^−1^ at calibration). TLC plates used were 20 × 20 cm silica-coated glass plates (Silica 60) with a concentration zone (Merck, Germany).

### Parasite maintenance and gametocyte purification

All parasites used in this study were derived from the *P. berghei* ANKA clone 2.34. For Ca^2+^ measurements, the clone 1.7.8 was used as previously described (Billker *et al*., 2004). Parasites were maintained in female NMRI mice (Charles River). This research adhered to the Principles of Laboratory Animal Care. The animal study was approved by the local animal use committees in compliance with European regulations and national legislation. Gametocytes were purified as described previously (Billker *et al*., 2004) with minor modifications. Mice were pre-treated with 0.1 ml phenylhydrazine (25 mg ml^−1^ in PBS) and infected 2 days later with 0.5–2 × 10^7^ parasites from frozen blood stocks. On day 4 p.i. 20 mg l^−1^ sulfadiazine in drinking water was applied to kill asexual stages. On day 6 p.i., mice were bled by cardiac puncture, the blood washed in gametocyte maintenance buffer (GMB: 137 mM NaCl, 4 mM KCl, 1 mM MgCl_2_, 1 mM CaCl_2_, 20 mM glucose, 20 mM Hepes, 4 mM sodium bicarbonate, 0.1% BSA, [pH 7.24–7.29]) and white blood cells were removed on CF11 cellulose (Whatman) columns. Gametocytes were purified on a 48% Nycodenz/GMB cushion [Nycodenz stock solution: 27.6% w/v Nycodenz in 5 mM Tris-HCl (pH 7.2), 3 mM KCl, 0.3 mM EDTA]. After purification gametocytes were resuspended in GMB and kept at 20°C and their purity examined on Giemsa stained blood films. On average, gametocytes were enriched to approximately 95% with contaminants mainly being late stage trophozoites (∼4%), few red blood cells and occasionally very few white blood cells.

### Gametocyte activation, exflagellation and Ca^2+^ measurements

All experiments were carried out at room temperature (20–26°C) unless otherwise indicated. For phospholipid measurements gametocytes were activated by transferring them to gametocyte activation medium (GAM), RPMI 1640 with 20 mM Hepes, 4 mM sodium bicarbonate, pH 8.0 containing 100 µM XA, unless otherwise stated. To count exflagellation events either 10 µl purified gametocytes were resuspended in 50 µl GAM or 3 µl of tail blood from an infected mouse were washed rapidly in 1 ml GMB, pelleted and resuspended in 30 µl GAM. A drop was then placed on a microscope slide and covered with a Vaseline rimmed coverslip. Exflagellation events were then counted at 400× magnification after 12–15 min. DNA synthesis during microgametogenesis was measured through incorporation of radioactive [^3^H]hypoxanthine as described elsewhere (Raabe *et al*., 2009). The luminometric Ca^2+^ assay was performed on enriched gametocytes exactly as described previously (Billker *et al*., 2004). Briefly, gametocytes were loaded with the luciferin, coelenterazine-fcp (Biotrend, Germany), for 30 min at 20°C, in loading buffer containing 1 mM EGTA, pH 7.25. Washed gametocytes were then auto-injected, one well at a time into 96-well plates containing test compounds and 100 µM XA or control solutions. Bioluminescence was counted in a Berthold Orion II luminometer. The presence of either 20 µM or 100 µM XA in the different activation media had no influence on the results and both concentrations result in maximal activation of gametocytes.

### Measurement of IP_3_ and PIP_2_

The Biotrak radioreceptor assay (GE Healthcare, product code TRK1000) was used, which determines IP_3_ in a sample by its ability to displace a [^3^H]IP_3_ radiotracer from a high affinity IP_3_ receptor protein (Palmer *et al*., 1989). Fifty microlitres of enriched gametocytes in GMB at 20°C (0.5–2 × 10^7^ cells) was activated by transfer into 150 µl GAM. Parasite development was stopped by addition of 200 µl ice cold 10% (v/v) 1 M perchloric acid. IP_3_ was extracted using freon/octylamide as described in the manufacturer's manual.

### ^32^P labelling and analysis of phosphoinositides by thin layer chromatography

A total of 3 × 10^8^ purified gametocytes were resuspended in 800 µl GMB containing the radioactive label ^32^P (final concentration depending on specific activity, usually 1 mCi) and incubated for 1.5–2 h at 20°C under agitation in an Eppendorf Thermomixer at 1000 r.p.m. Following three GMB washes in the presence of 10 mM cold phosphate, cells were resuspended and incubated at 20°C, 1000 r.p.m. for another 3 min to purge the cells of radioactive ATP. Gametocytes were then activated by addition of XA or A23187 and the reactions stopped by transferring aliquots (200 µl) into screw cap glass tubes containing 1.2 ml ice cold methanol. The following solvents were added with intermittent vortexing steps: 600 µl chloroform, 20 µl HCl (12 M), 600 µl chloroform, 600 µl KCl (2 M). Following centrifugation (3000 r.p.m., 20°C, 5 min) the lower phase was transferred to a new glass tube. The upper phase was washed by adding 2 ml chloroform and lower phases pooled. The solvent of the pooled fractions was evaporated under N_2_ flow at room temperature. For loading on a TLC plate, lipids were resuspended in 100 µl chloroform/methanol (2:1, v/v) and 10–20 µl applied onto the concentration zone of a TLC plate, which had previously been incubated for 15 min in oxalate solution [1% Potassium-Oxalate, 2 mM EDTA)/(Methanol) 1/1 (v/v)] and heat activated at 100°C for 1 h. TLC was performed with [CHCl_3_/CH_3_COCH_3_/CH_3_OH/ CH_3_COOH/H_2_O, 80/30/26/24/14 (v/v)]. TLC plates were revealed using a phosphoimaging scanner. The quantification of PIP and PIP_2_ was done by either scraping off the silica of bands of interest and analysis in a Beckman Coulter Multi-Purpose Scintillation Counter, or by using the Image Quant v5.2 software to obtain relative intensity levels.

### Cloning of CFP–PH–YFP

The PH domain of human phospholipase Cδ1 (hPLCδ1) fused to CFP and YFP was isolated from a pcDNA3.1(+)-based plasmid containing the CYPHR fusion protein (Violin *et al*., 2003) as a HindIII/XbaI fragment and subsequently blunted. The *P. berghei* expression vector pDEFGFPM3A encodes the green fluorescent protein (GFP) and is equivalent to MR4 reagent MRA-786 (pL0017) differing from the pPbGFP_CON_ (Franke-Fayard *et al*., 2004) only by the presence of an additional XbaI site immediately following the stop codon of *gfp*. The *gfp* coding sequence was removed by a BamHI/XbaI digest, the vector blunted and the CFP–PH–YFP sequence inserted. Correct insertion and the sequence of CFP–PH–YFP were confirmed by sequencing. Plasmid pDEFGFPM3A was used as control construct without a PH domain. Parasites harbouring the plasmids as episomes were generated as described (Janse *et al*., 2006) by electroporation of enriched schizonts followed by selection with pyrimethamine in the drinking water of infected mice.
